# Status of HIV Case-Based Surveillance Implementation — 39 U.S. PEPFAR-Supported Countries, May–July 2019

**DOI:** 10.15585/mmwr.mm6847a2

**Published:** 2019-11-29

**Authors:** Joshua R. Holmes, Thu-Ha Dinh, Nasim Farach, Eric-Jan Manders, James Kariuki, Daniel H. Rosen, Andrea A. Kim, PEPFAR HIV Case-Based Surveillance Study Group, PEPFAR HIV Case-based Surveillance Study Group, Alean Frawley, Patrick Tema, Kristen L. Hess, Khuteletso Bagapi, Penh Sun Ly, Vanthy Ly, Rachel Albalak, Varough Deyde, Tyson Volkmann, Martine Chase, Rhonda A. Moore, Sasha E. Walrond, Avery Hinds, Keven Antoine, Nicola Skyers, Jose Manuel Rodas, Sandra I. Juarez, Tomasa Sierra Pineda, José Salvador Sorto, Maria Mastelari, Manuel Sagastume, Ingrid Castillo, Luz Maria Romero, Enrique Beteta, Aka-Camara Aoua, G. Laissa Ouedraogo, Legre Roger Lobognon, Rinaldy Capellán, Luis Bonilla, Jacob Dee, Rogers Galaxy Ngalamulume, Denis Yoka Ebengo, Caroline Ryan, Munyaradzi Pasipamire, Beyene Moges, Frehywot Eshetu, Ayalew A. Haile, Silas Quaye, Valerie Pelletier, Joelle Deas Van Onacker, Timbila Jean Baptiste Koama, Leonard Kingwara, Faith N. Ngari, Catherine Ngugi, Anthony Waruru, Joseph L. Barker, Margaret Ndisha, Peter Young, Phouthone Southalack, Bouathong Simanovong, Douangchanh Xaymounvong, Martha Conkling, Refiloe Mpholo, Tigest Mekonnen, Linda Mattocks, Evelyn Kim, Subrat Das, Mamadou B. Traore, Sory Traore, Kristen Heitzinger, Maria Rein, Peter Kerndt, Adam Wolkon, Michael de Klerk, Nicholus Mutenda, Charles Nzelu, Ibrahim Dalhatu, Stacie Greby, Ibrahim Jahun, Mukhtar Ahmed, Victor Sebastian, Ademola Oladipo, Matthias Alagi, Moyosola Bamidele, Mustapha Bello, Henry Debem, Raphael Akpan, Aminu Yakubu, Ayodele Fagbemi, Nguhemen Tiger, Ifunanya Mgbakor, Ibrahim Dangana, Abel Yamba, Poruan Temu, Peniel Boas, Gene MacDonald, Janise Richards, Placidie Mugwaneza, Mboup Souleymane, Astou Guèye Gaye, Safiatou Thiam, Moussa Sarr, Mireille Cheyip, Zukiswa Edna Pinini, Sarah Porter, Shirley Nkone, Richard Lino Loro Lako, Moses Mutebi Nganda, Sudhir Bunga, Alex Bolo, George S. Mgomella, Jeremiah Mushi, Zaharani Kalungwa, Suvimon Tanpradech, Kunjanakorn Phokhasawad, Thitipong Yingyong, Herbert Kiyingi, Muramuzi Bangizi Emmy, Edgar Kansiime, Musenge Kenneth, Roksolana Kulchynska, Ihor Kuzin, Nataliya Podolchak, Ezra J. Barzilay, Violetta Martsynovska, Abu S. Abdul-Quader, Vo Hai Son, Nguyen Tuan Anh, Suilanjo Sivile, Andrew Banda, Stanley Kamocha, Elizabeth Gonese, Brian Kumbirai Moyo, Kelsey Mirkovic

**Affiliations:** 1 Division of Global HIV and TB, Center for Global Health, CDC.; CDC-Angola; Ministry of Health, Botswana; CDC-Botswana; CDC-Botswana; Ministry of Health, Cambodia; CDC-Cambodia; CDC-Cambodia; CDC-Caribbean Regional Office; CDC-Caribbean Regional Office; CDC-Caribbean Regional Office; Ministry of Public Health, Guyana; Ministry of Public Health, Guyana; Ministry of Health, Trinidad and Tobago; Ministry of Health, Trinidad and Tobago; Ministry of Health and Wellness, Jamaica; CDC-Central America Regional Office; CDC-Central America Regional Office; Ministry of Health, Honduras; Ministry of Health, El Salvador; Ministry of Health, Panama; Ministry of Health, Guatemala; Ministry of Health, Guatemala; Unversidad del Valle de Guatemala; Ministry of Health, Nicaragua; Ministry of Health and Public Hygiene, Côte d’Ivoire; CDC-Côte d’Ivoire; CDC-Côte d’Ivoire; Direccíon General de Epidemiología, Dominican Republic; CDC-Dominican Republic; CDC-Democratic Republic of the Congo; CDC-Democratic Republic of the Congo; Ministry of Public Health, Democratic Republic of the Congo; CDC-Eswatini; CDC-Eswatini; Ethiopian Public Health Institute; CDC-Ethiopia; International Centers for AIDS Care and Treatment Programs, Ethiopia; CDC-Ghana; CDC-Haiti; Ministère de la Santé Publique et; de la Population, Haiti; CDC-Haiti; Ministry of Health, Kenya; Ministry of Health, Kenya; Ministry of Health, Kenya; CDC-Kenya; CDC-Kenya; CDC-Kenya; CDC-Kenya; Ministry of Health, Laos;; Ministry of Health, Laos; CDC-Laos; CDC-Lesotho; CDC-Lesotho; CDC-Malawi; CDC-Malawi; CDC-Malawi; CDC-Mali; CDC-Mali; Ministry of Health, Mali; CDC-Mozambique; CDC-Mozambique; CDC-Mozambique; CDC-Namibia; CDC-Namibia; Ministry of Health and Social Services, Namibia; Federal Ministry of Health, Nigeria; CDC-Nigeria; CDC-Nigeria; CDC-Nigeria; CDC-Nigeria; CDC-Nigeria; CDC-Nigeria; CDC-Nigeria; CDC-Nigeria; CDC-Nigeria; CDC-Nigeria; CDC-Nigeria; CDC-Nigeria; CDC-Nigeria; CDC-Nigeria; CDC-Nigeria; CDC-Nigeria; CDC-Papua New Guinea; CDC-Papua New Guinea; National Department of Health, Papua New Guinea; CDC-Rwanda; CDC-Rwanda; Rwanda Biomedical Center; Institut de Recherche en Santé, de Surveillance Epidémiologique et de Formations, Senegal; Institut de Recherche en Santé, de Surveillance Epidémiologique et de Formations, Senegal; Conseils National de Lutte Contre le SIDA, Senegal; Westat, Senegal; CDC-South Africa; National Department of Health, South Africa; CDC-South Africa; CDC-South Africa; Ministry of Health, South Sudan; Mbarara University of Science and Technology, Uganda; CDC-South Sudan; CDC-South Sudan; CDC-Tanzania; Ministry of Health, Community Development, Gender, Elderly and Children, Tanzania; CDC-Tanzania; CDC-Thailand; CDC-Thailand; Ministry of Public Health, Thailand; CDC-Uganda; Ministry of Health, Uganda; School of Public Health, Makerere University, Uganda; CDC-Uganda; CDC-Ukraine; Public Health Center of the Ministry of Health, Ukraine; CDC-Ukraine; CDC-Ukraine; Public Health Center of the Ministry of Health, Ukraine; CDC-Vietnam; Ministry of Health, Vietnam; CDC-Vietnam; Ministry of Health, Zambia; University of Zambia; CDC-Zambia; CDC-Zimbabwe; Ministry of Health and Child Care, Zimbabwe; CDC-Zimbabwe.

Human immunodeficiency virus (HIV) case-based surveillance (CBS) systematically and continuously collects available demographic and health event data (sentinel events*) about persons with HIV infection from diagnosis and, if available, throughout routine clinical care until death, to characterize HIV epidemics and guide program improvement (*1*,*2*). Surveillance signals such as high viral load, mortality, or recent HIV infection can be used for rapid public health action. To date, few standardized assessments have been conducted to describe HIV CBS systems globally (*3*,*4*). For this assessment, a survey was disseminated during May–July 2019 to all U.S. President’s Emergency Plan for AIDS Relief (PEPFAR)–supported countries with CDC presence^†^ (46) to describe CBS implementation and identify facilitators and barriers. Among the 39 (85%) countries that responded,^§^ 20 (51%) have implemented CBS, 15 (38%) were planning implementation, and four (10%)^¶^ had no plans for implementation. All countries with CBS reported capturing information at the point of diagnosis, and 85% captured sentinel event data. The most common characteristic (75% of implementation countries) that facilitated implementation was using a health information system for CBS. Barriers to CBS implementation included lack of country policies/guidance on mandated reporting of HIV and on CBS, lack of unique identifiers to match and deduplicate patient-level data, and lack of data security standards. Although most surveyed countries reported implementing or planning for implementation of CBS, these barriers need to be addressed to implement effective HIV CBS that can inform the national response to the HIV epidemic.

In 2017, CDC initially assessed clinical surveillance among CDC PEPFAR-supported countries (*4*). The survey was revised in 2019 with feedback from stakeholders** to focus on CBS and client-level HIV health information system as they relate to CBS. Research Electronic Data Capture (REDCap) (*5*,*6*), an electronic data management tool hosted at CDC and distributed to each PEPFAR-supported CDC country or regional office (representing 46 countries) during May–July 2019 was used to collect responses. CDC country office representatives were asked to complete the survey in partnership with local government officials (ministries of health and implementing partners). The protocol for this activity was reviewed in accordance with CDC human research protection procedures and was determined to be nonresearch.

The survey included questions on functional requirements, security measures, national policies and guidelines, and barriers for CBS implementation (Supplementary table, https://stacks.cdc.gov/view/cdc/82569). Answers were reported based on the country’s CBS status (currently implementing CBS, planning to implement, or not planning to implement). In one country, respondents reported uncertainty about future CBS implementation, so this country was grouped with countries not planning implementation. Functional requirements and facilitators included using unique identifiers^††^ to link and deduplicate patient data, having national policies for including HIV infection as a notifiable disease, and reporting unique cases of HIV infection and sentinel events to a public health program for surveillance. Barriers to implementation included lack of policies related to CBS, data security, confidentiality, and privacy of HIV information; criminalization laws; and stigmatization and criminalization of populations at greatest risk for HIV infection.^§§^ Additional questions assessing implementation barriers were asked of countries that were not planning to implement CBS.

Several questions applied only to countries that had implemented or were planning to implement CBS. These included whether the system captured (or will capture) date of diagnosis of HIV infection and subsequent sentinel events data and security measures for transmitting paper-based data and for transmitting and storing electronic data. Implementing countries also reported information on whether they were using a health information system for CBS.

Among the 46 PEPFAR-supported countries surveyed, 39 (85%) completed the survey. Despite multiple follow-up attempts, seven countries did not complete the assessment. Skip patterns in the survey resulted in some questions not being asked of all responding countries. Descriptive statistics for aggregated and country-level responses^¶¶^ for primary variables were performed using SAS statistical software (version 9.4; SAS Institute).

Overall, 20 (51%) countries reported implementing CBS, 15 (38%) were planning implementation, three (8%) were not planning implementation, and one (3%) was unsure of future implementation (Table 1). Implementation status substantially varied among continents. All surveyed countries in the regions*** of Americas (11) and Europe (one) reported having implemented CBS. Among five surveyed countries in Asia, three (Papua New Guinea, Thailand, and Vietnam) had implemented CBS, and two (Cambodia and Laos) were planning implementation, whereas among 22 countries in sub-Saharan Africa, only five (Botswana, Ethiopia, Rwanda, Senegal, and Zimbabwe) reported having implemented CBS. Among the remaining 17 sub-Saharan African countries, 13 were planning implementation, three had no plans for implementation, and one was unsure about plans for implementing CBS.

**TABLE 1:** Status of implementation of case-based surveillance for human immunodeficiency virus infection in 39 countries supported by the U.S. President’s Emergency Plan for AIDS Relief, May–July 2019

Region*/Country	Implementing	Planning implementation	Not planning implementation	Unsure^†^
**Africa (n = 22)**				
Angola	—	—	Yes	—
Botswana	Yes	—	—	—
Côte d’Ivoire	—	Yes	—	—
DRC	—	—	Yes	—
Eswatini	—	—	—	Yes
Ethiopia	Yes	—	—	—
Ghana	—	Yes	—	—
Kenya	—	Yes	—	—
Lesotho	—	Yes	—	—
Malawi	—	Yes	—	—
Mali	—	—	Yes	—
Mozambique	—	Yes	—	—
Namibia	—	Yes	—	—
Nigeria	—	Yes	—	—
Rwanda	Yes	—	—	—
Senegal	Yes		—	—
South Africa	—	Yes	—	—
South Sudan	—	Yes	—	—
Tanzania	—	Yes	—	—
Uganda	—	Yes	—	—
Zambia	—	Yes	—	—
Zimbabwe	Yes	—	—	—
**Americas (n = 11)**				
Brazil	Yes	—	—	—
Dominican Republic	Yes	—	—	—
El Salvador	Yes	—	—	—
Guatemala	Yes	—	—	—
Guyana	Yes	—	—	—
Haiti	Yes	—	—	—
Honduras	Yes	—	—	—
Jamaica	Yes	—	—	—
Nicaragua	Yes	—	—	—
Panama	Yes	—	—	—
Trinidad and Tobago	Yes	—	—	—
**Asia (n = 5)**				
Cambodia	—	Yes	—	—
Laos	—	Yes	—	—
Papua New Guinea	Yes	—	—	—
Thailand	Yes	—	—	—
Vietnam	Yes	—	—	—
**Europe (n = 1)**				
Ukraine	Yes	—	—	—
**Total (N = 39)**	**20**	**15**	**3**	**1**

**Abbreviations:** AIDS = acquired immunodeficiency syndrome; DRC = Democratic Republic of the Congo.

* World Health Organization (WHO) regions were used to group countries in the Americas, Europe, and Africa; countries in Asia were grouped into a single region, rather than the two regions (Southeast Asia and Western Pacific) designated by WHO.

^†^ The “unsure” and “not planning implementation” categories are reported separately here but were combined for analyses because of small sample size.

Among the 20 implementing countries, all collect the date of diagnosis of HIV infection, and 17 (85%) collect sentinel event data; however, only 10 of these countries reported using a unique identifier for linking and deduplicating patient-level data (Table 2). An electronic health information system was used by 15 (75%) countries that have implemented CBS. Among the 18 implementing countries asked about electronic-based security measures, all reported having one or more such measures for transmitting data (if applicable), and 19 of 20 had such measures for storing data. Among 16 countries implementing paper-based CBS,^†††^ 14 reported adopting one or more security measures.

**TABLE 2:** Human Immunodeficiency virus (HIV) case-based surveillance functional requirements, security measures, national policies and guidelines, and barriers, by implementation status,*39^†^ countries supported by the U.S. President’s Emergency Plan for AIDS Relief, May–July 2019

Case-based surveillance characteristics	Case-based surveillance implementation status (no. of countries) %^§^ (no./total no.^¶^)			
	Implementing (20)	Planning implementation (15)	Not planning implementation** (4)	Total (39)
**Functional requirements**				
Use of unique identifiers^††^	50 (10/20)	27 (4/15)	0 (0/4)	**36 (14/39)**
Captures (or will capture) diagnosis and date of diagnosis	100 (20/20)	87 (13/15)	—^§§^	**94 (33/35)**
Captures (or will capture) ≥1 sentinel event^¶¶^ with date	85 (17/20)	73 (11/15)	—	**80 (28/35)**
Health information system integrated into case-based surveillance***	75 (15/20)	—	—	**75 (15/20)**
**Security measures**				
Paper-based^†††^	88 (14/16)	88 (7/8)	—	**88 (21/24)**
Electronic-based: storage of data^§§§^	95 (19/20)	93 (14/15)	—	**94 (33/35)**
Electronic-based: transmission of data^¶¶¶^	100 (18/18)	100 (14/14)	—	**100 (32/32)**
**National policies and guidelines**				
HIV infection is a notifiable condition	80 (16/20)	33 (5/15)	0 (0/4)	**54 (21/39)**
Mandated reporting of subsequent health events for diagnosed HIV-positive cases****	63 (10/16)	40 (2/5)	—	**57 (12/21)**
Mandated security measures for data storage	85 (17/20)	67 (10/15)	—	**77 (27/35)**
Mandated reporting of HIV infection to a public health surveillance system	85 (17/20)	40 (6/15)	0 (0/4)	**59 (23/39)**
**Barriers to implementation and maintenance**				
No national policy/guidance for case-based surveillance	15 (3/20)	67 (10/15)	75 (3/4)	**41 (16/39)**
No policies for data security, confidentiality, or privacy of HIV information	20 (4/20)	7 (1/15)	25 (1/4)	**15 (6/39)**
HIV criminalization laws	10 (2/20)	7 (1/15)	0 (0/4)	**8 (3/39)**
Stigmatization/Criminalization of populations at high risk^††††^	30 (6/20)	40 (6/15)	100 (4/4)	**41 (16/39)**
No funding	—	—	50 (2/4)	**50 (2/4)**
No dedicated human resources	—	—	50 (2/4)	**50 (2/4)**
Not a current priority	—	—	25 (1/4)	**25 (1/4)**
No perceived need	—	—	0 (0/4)	**0 (0/4)**

**Abbreviation:** AIDS = acquired immunodeficiency syndrome.

* Implementing countries include those that reported having an HIV case-based surveillance system in which individual-level information on diagnosed HIV cases is reported for surveillance purposes; planning countries include those that reported having plans to implement case-based surveillance; and the not planning category includes countries that reported not having plans to implement case-based surveillance.

^†^ Angola, Botswana, Brazil, Cambodia, Côte d’Ivoire, Democratic Republic of the Congo, Dominican Republic, El Salvador, Eswatini, Ethiopia, Ghana, Guatemala, Guyana, Haiti, Honduras, Jamaica, Kenya, Laos, Lesotho, Mali, Malawi, Mozambique, Namibia, Nicaragua, Nigeria, Panama, Papua New Guinea, Rwanda, Senegal, South Africa, South Sudan, Tanzania, Thailand, Trinidad and Tobago, Uganda, Ukraine, Vietnam, Zambia, and Zimbabwe.

^§^ Column percentages might not sum to 100% because of rounding.

^¶^ Total number might vary based on number of countries to which each question was asked.

** One country reported not having case-based surveillance and was unsure about future implementation. Because of small sample size, this country was grouped with those that reported having no plans to implement case-based surveillance.

^††^ Unique identifiers include health identifier, passport number, driver license, biometrics, program specific identifier (e.g., antiretroviral therapy number), civil identity card, and pseudo-identifier that can be used to connect and deduplicate patient data across facilities.

^§§^ Dashes indicate that some questions were not asked for countries based on self-reported status of case-based surveillance implementation.

^¶¶^ Sentinel events data include various events throughout medical care for a client with diagnosed HIV infection, such as HIV recency status (recent or long-term infection at time of diagnosis), clinical laboratory values such as CD4 count and viral load, change in antiretroviral therapy regimens, and death.

*** Countries were asked if they reported using health information systems for case-based surveillance.

^†††^ Among countries reporting paper-based abstraction of case-based surveillance data and/or using courier for sending paper case report forms to the above-site level (implementing countries, n = 16; planning countries, n = 8). Paper-based security measures include at least one of the following: forms kept in a secure and locked location or record retention policies.

^§§§^ Electronic-based security measures include one of more of the following steps: encryption of data; software barrier; limited personnel access; multifactor authentication; periodic password changes and/or complex passwords; and laws, policies, guidelines, or standard operating procedures mandating security.

^¶¶¶^ Among countries reporting electronic transmission of case-based surveillance data (implementing countries, n = 18; planning countries, n = 14). Electronic-based security measures include one of more of the following steps: encryption of data; software barrier; limited personnel access; multifactor authentication; periodic password changes and/or complex passwords; and laws, policies, guidelines, or standard operating procedures mandating security.

**** Among countries in which HIV infection is a nationally notifiable condition (implementing countries, n = 14; planning countries, n = 7).

^††††^ Groups that have high risk of HIV infection, including female sex workers, men who have sex with men, persons who inject drugs, transgender persons, and persons incarcerated.

Among the 15 countries planning to implement CBS, 13 planned to collect date of diagnosis data, and 11 planned to collect sentinel event data with date of events (Table 2). Four countries planning implementation of CBS have the capability to use unique identifiers to link and deduplicate patient-level data. Similar to countries that have already implemented CBS, all of the 14 countries planning to implement reported planning for security measures for transmitting data (if applicable), 14 of 15 reported planning for security measures for storing data, and seven of eight reported planning to implement paper-based surveillance reported planning for security measures (Table 2).

Many countries reported barriers to implementation of CBS. Stigmatization and criminalization of populations at high risk of HIV infection were reported by six of 20 countries that had implemented CBS, by six of 15 that were planning implementation, and by all four that were not planning to implement. Ten of 15 countries planning to implement reported the lack of national policy/guidance for CBS as an important barrier to implementation. Barriers reported by countries not planning to implement CBS included lack of funding and dedicated human resources. HIV was a nationally notifiable condition in 16 of 20 implementing countries, in five of 15 countries planning to implement CBS, and in none of the countries that did not have plans to implement CBS (Table 2).

## Discussion

Although 35 (90%) of 39 PEPFAR-supported countries that responded to the survey have implemented HIV CBS or are planning implementation, barriers to implementation were identified in most countries, including absence of policies related to HIV reporting and CBS, nonuniversal adoption of security measures for electronic-based and paper-based systems, lack of unique identifiers, and no collection of postdiagnosis sentinel event data. The fact that only half of countries implementing CBS use a unique identifier to match and deduplicate data highlights a need to improve understanding of the functional requirements of CBS. Ministries of health can request partners with surveillance, informatics, and policy expertise to assist in identifying barriers to implementing effective HIV CBS and in developing solutions.

Among the 39 participating countries, 22 (56%) were in sub-Saharan Africa; however, only 23% of these countries had implemented CBS. This finding might be partly explained by the region’s high HIV prevalence, less developed health information system infrastructure, and fewer resources compared with countries with lower HIV prevalence or an epidemic among specific populations, such as those in the Americas, Asia, and Europe (*7*). Because HIV is a notifiable condition in most implementing countries, national policy changes could support CBS implementation. Implementing CBS for public health is an important policy consideration for all PEPFAR-supported countries (*2*); however, the fact that many countries have not yet implemented CBS underscores the need for increased efforts to address policy barriers and gaps in technical infrastructure so that comprehensive HIV CBS systems that can inform national responses to the HIV epidemic can be implemented.

These findings are subject to at least four limitations. First, several countries did not complete the survey despite multiple follow-up attempts; thus, these results might not be representative of all PEPFAR-supported countries. Second, this assessment might not have identified all potential facilitators and barriers for CBS implementation. Third, because the survey was self-administered, the questions might have been interpreted differently by different respondents. Finally, although persons familiar with the country’s HIV surveillance systems were requested to complete the survey, not all responses were verified and were subject to reporting bias; in some cases, some responses were confirmed through follow-up communication with the respondent.

Despite these limitations, this is the first comprehensive global assessment of CBS implementation in PEPFAR-supported countries. CBS is an effective system for countries to monitor their HIV epidemics in real time and to better inform responses. The assessment identified important barriers that need to be addressed to implement CBS effectively. Moving forward, annual deployments of this assessment can help monitor countries’ progress toward successful CBS implementation.

**Summary**

**What is already known on this topic?**

Human immunodeficiency virus (HIV) case-based surveillance continuously and systematically monitors HIV-positive patients throughout their clinical care and facilitates rapid public health action.

**What is added by this report?**

Among 39 surveyed countries supported by the U.S. President’s Emergency Plan for AIDS Relief, 20 had implemented case-based surveillance, 15 were planning implementation, and four were not planning implementation. Challenges for most countries, particularly those in sub-Saharan Africa, include need for unique identifiers to link data across systems, supportive national policy environments, and data security standards.

**What are the implications for public health practice?**

Enhanced efforts are needed to address policy barriers and gaps in technical infrastructure to implement comprehensive HIV case-based surveillance that can inform national response to the HIV epidemic.

## Footnotes

* Sentinel events include various events throughout medical care for a client with diagnosed HIV infection, such as HIV recency status (recent or long-term infection at time of diagnosis), clinical laboratory values such as CD4 count and viral load, change in antiretroviral therapy regimens, and death.

† PEPFAR-supported countries include Angola, Barbados, Botswana, Brazil, Burma, Cambodia, Cameroon, Côte d’Ivoire, Democratic Republic of the Congo, Dominican Republic, El Salvador, Eswatini, Ethiopia, Ghana, Guatemala, Guyana, Haiti, Honduras, India, Jamaica, Kazakhstan, Kenya, Kyrgyzstan, Laos, Lesotho, Malawi, Mali, Mozambique, Namibia, Nicaragua, Nigeria, Panama, Papua New Guinea, Rwanda, Senegal, South Africa, South Sudan, Tajikistan, Tanzania, Thailand, Trinidad and Tobago, Uganda, Ukraine, Vietnam, Zambia, and Zimbabwe.

§ No data for Barbados, Burma, India, Cameroon, Kazakhstan, Kyrgyzstan, or Tajikistan.

¶ Implementing countries include those that reported having an HIV case-based surveillance system in their country at any scale (e.g., pilot or national) in which individual-level information on diagnosed HIV cases are reported for surveillance purposes; planning countries include those that reported planning to implement case-based surveillance; the not planning category includes countries that reported not having plans to implement case-based surveillance; and the unsure country reported uncertainty on future implementation.

** World Health Organization (WHO), Joint United Nations Programme on HIV/AIDS, Global Fund, ministries of health, and CDC country offices.

†† Unique identifiers include health identifier, passport number, driver license, biometrics, program-specific identifier (e.g., antiretroviral therapy number), civil identity card, and pseudo-identifier.

§§ Includes female sex workers, men who have sex with men, persons who inject drugs, transgender persons, and persons incarcerated.

¶¶ The country-level indicator is the current state of case-based surveillance implementation (implementing, planning implementation, not planning implementation, and unsure).

*** WHO regions were used to group countries in the Americas, Europe, and Africa. Countries in Asia were grouped into a single region, rather than the two regions (Southeast Asia and Western Pacific) designated by WHO.

††† Among countries reporting paper-based abstraction of case-based surveillance data or using a courier for sending paper case report forms to the above-site level (n = 16).
